# Progress in the Surface Functionalization of Selenium Nanoparticles and Their Potential Application in Cancer Therapy

**DOI:** 10.3390/antiox11101965

**Published:** 2022-09-30

**Authors:** Wanwen Chen, Hao Cheng, Wenshui Xia

**Affiliations:** 1 State Key Laboratory of Food Science and Technology, School of Food Science and Technology, Jiangnan University, Wuxi 214122, China; 2 Collaborative Innovation Center of Food Safety and Quality Control in Jiangsu Province, Jiangnan University, Wuxi 214122, China

**Keywords:** selenium nanoparticles, surface functionalization, stability, targeting delivery, anticancer activity

## Abstract

As an essential micronutrient, selenium participates in numerous life processes and plays a key role in human health. In the past decade, selenium nanoparticles (SeNPs) have attracted great attention due to their excellent functionality for potential applications in pharmaceuticals. However, the utilization of SeNPs has been restricted by their instability and low targeting ability. Since the existing reviews mainly focused on the applications of SeNPs, this review highlights the synthesis of SeNPs and the strategies to improve their stability and targeting ability through surface functionalization. In addition, the utilization of functionalized SeNPs for the single and co-delivery of drugs or genes to achieve the combination of therapy are also presented, with the emphasis on the potential mechanism. The current challenges and prospects of functionalized SeNPs are also summarized. This review may provide valuable information for the design of novel functionalized SeNPs and promote their future application in cancer therapy.

## 1. Introduction

Cancer involves multistep processes, including uncontrolled cell division, tumor development, invasion, and metastasis. The complexity, diversity, and heterogeneity of tumors lead to one in six deaths worldwide [1]. Although a variety of conventional anticancer drugs have been developed for the improvement of patient survival, the poor water solubility, nonspecific selectivity, drug resistance, as well as severe side effects seriously undermine their therapeutic potential for treatment [2]. It is well known that cancer cells fail to adapt to any additional oxidative burst due to the aberrant metabolism. Thus, the use of a redox modulator provides a therapeutic window for successful anticancer therapy [3]. Selenium is a trace element that has been declared by the World Health Organization (WHO) in 1973 [4]. It is a basic component of 25 selenoproteins and enzymes, such as glutathione peroxidases, thioredoxin reductases, selenoprotein P, selenoprotein F, selenoprotein S, and selenoprotein M, playing a key role as a redox modulator in maintaining intracellular redox balance [5,6]. Although inorganic and organic selenium presents various oxidation states of −2, 0, +4, and +6 with prooxidant characters that have demonstrated potential anticancer activity [7], there is a narrow therapeutic window between anticancer efficacy and toxicity [8]. Thus, the development of novel selenium species has become a top priority.

The emergence of nanotechnology has provided novel strategies for fabricating nanomaterials with unique physical, chemical, and biological properties due to their nano-size [9]. Selenium nanoparticles (SeNPs) possess higher anticancer activity, biocompatibility, and lower toxicity than inorganic or organic selenium compounds, attracting increasing attention as agents for cancer prevention or as nanocarriers of drugs/genes for cancer therapy. The potential antitumor mechanisms of SeNPs have been demonstrated as follows: (i) The induction of apoptosis via intrinsic or mitochondrial-dependent pathway; (ii) The induction of apoptosis through extrinsic or death receptor pathway; (iii) The induction of cell-cycle arrest; (iv) The enhancement of immunity [10]. The most frequently applied approach for the synthesis of SeNPs was the utilization of chemical agents or biological organisms to reduce oxidized forms of selenium to the elemental form, accompanied by several physical methods [11]. However, bare SeNPs with valence state zero are highly unstable in an aqueous solution, which might be attributed to the high surface energy, leading to the aggregation of SeNPs, resulting in lower bioactivity, further limiting their practical application [12]. Thus, many endeavors have been made on decorating or functionalizing SeNPs using biomolecules (e.g., polysaccharides, proteins, or phenols) to improve their stability. The stability and potential biological activity of SeNPs are mainly related to the functional groups bound to them, selenium concentration, as well as the nano range size [13]. In addition, although SeNPs are enabled to accumulate inside the tumor through enhanced permeability and retention effect (EPR) characteristics, the uptake efficiency was almost less than 1%. Conjugation of SeNPs with ligands (e.g., folic acid (FA), peptides, antibodies, or aptamers) not only leads to the enhancement of NPs internalization through receptor-mediated endocytosis but also facilities the NPs onto the specific sites to further improve the biological activity [2]. Moreover, the functionalized SeNPs have shown great potential as carriers of chemotherapeutic agents or genes including 5-Fluorouracil (5-Fu), doxorubicin (DOX), and small interfering RNA (siRNA), as well as curcumin, to achieve synergistic anticancer activity and overcome multidrug resistance [11].

**Figure 1:**
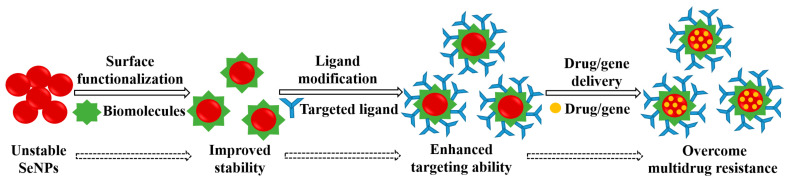
Illustrations of emphasized details in this review.

Although several reviews related to SeNPs have been reported, most of them mainly focused on the various functions and applications of SeNPs. The instability and low targeting ability are the key factors that restrict the actual applications of SeNPs. Thus, this review focuses on the current strategies for the chemical synthesis of SeNPs to improve their stability. The methodologies for the preparation of active-targeted SeNPs to enhance the cellular uptake efficiency are also discussed. We further highlight the application of functionalized SeNPs as nanocarriers for the single or co-delivery of chemotherapeutic agents or genes, and address the main anticancer mechanism of functionalized SeNPs in cancer therapy (Figure 1).

## 2. Synthesis, Surface Functionalization, and Stability of SeNPs

### 2.1. Synthesis of SeNPs

Colloidal SeNPs can be synthesized through physical, biological, or chemical methods depending on the desired application. (i) The physical methods include microwave irradiation [14], pulsed laser ablation [15], vapor deposition [16], and photocatalytic synthesis [17]. Although the physical methods present several advantages, such as rapid reaction time and enhanced reaction rates, it has been increasingly disfavored due to the high energy conditions with cost-ineffectiveness [18,19]. (ii) The biosynthesis of SeNPs by using bioorganisms, such as yeast, bacteria, fungi, as well as plants for the reduction in SeO_3_^2−^ to SeNPs [20]. It has the advantages of high yielding, cost-effectiveness, and sound biomedical nature. However, it suffers the drawbacks such as the long transformation time and complex procedures for purification [21]. (iii) To cope with these issues, the chemical synthesis of SeNPs is gaining more attention and is recommended as an alternative strategy. Briefly, reducing agents were used to reduce the selenium salt to the element selenium. It is a relatively simple, effective, and time-saving method, termed a “one-pot synthesis”, which could easily control the size, morphology, and surface chemistry of nanoparticles, improve the efficiency of the reaction, bypass complex purification steps, and achieve favorable biomedical functionality.

### 2.2. Surface Functionalization of SeNPs

**Figure 2:**
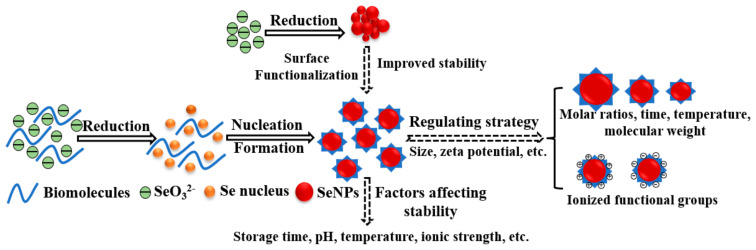
Schematic diagram of the formation process of biomolecules functionalized SeNPs.

Chemical synthesis is the most widely applied method for the preparation of SeNPs using sodium selenite, selenious acid, or selenium dioxide as precursors and ascorbic acid, sodium borohydride, or hydrazine hydrate as reducing agents. Unfortunately, SeNPs are highly unstable and are easily prone to aggregation and transform into inert forms in an aqueous medium [22]. Surface functionalization is an essential tool to promote dispersity and reduce the nonspecific interactions between the nanoparticles and other components, further improving the stability of SeNPs in physiological media. In addition, functionalization with a suitable stabilizer can not only modulate the physicochemical properties of SeNPs but also reduce their toxicity and enhance bioavailability [23]. Here we highlight the strategies for the functionalization of SeNPs with biomolecules to improve their stability (Figure 2). The reaction conditions, size, stability conditions tested, and stability of various biomolecules functionalized SeNPs were summarized in Table 1.

#### 2.2.1. Polysaccharides Functionalized SeNPs

Polysaccharides are ubiquitous biological macromolecules that are envisaged as promising substitutes owing to their biocompatibility, biodegradability, low toxicity, availability, and active functional groups (such as -OH, -COO^−^, -SO_3_O^−^, -NH_3_^+^), which could facilitate the controllable fabrication of nanocomposites [24]. Polysaccharides can not only be used as templates for the growth of nanoparticles but also prolong the residence time and act synergistically to enhance biological activity [25]. Moreover, several polysaccharides can be used both as the surface decorator and the reducing agent for the facile synthesis of highly uniform SeNPs [26]. Thus, numerous researchers have made efforts to fabricate SeNPs with natural polysaccharides to overcome the limitation of SeNPs and endow desired properties. The main process of most polysaccharides-capped SeNPs can be illustrated as follows: sodium selenite is mixed with polysaccharides first, followed by the addition of a reducing agent to form SeNPs in the presence of polysaccharides [4]. From now on, dialysis against ultrapure water for overnight or longer is the commonly used method to purify SeNPs by eliminating unreacted substances. However, this procedure prolongs synthesis time. One study using centrifugation replaced dialysis for the exploration of a timeless and efficient way to purify SeNPs. Although a decrease in selenium concentration occurred after centrifugation and resuspension, the resuspended SeNPs@CS also exhibited a relatively uniform and monodispersed spherical structure as before [27]. The proposed mechanism for the formation of polysaccharides functionalized SeNPs can be illustrated as follows. (i) SeO_3_^2−^ was initially adsorbed on the molecular chains of polysaccharides through electrostatic interactions or hydrogen bonds (O-H⋯Se) to form an intermediate. (ii) Anionic SeO_3_^2−^ could be reduced to elemental Se in situ by reducing agents. (iii) With an increasing amount of elemental Se, they would agglomerate into the Se nucleus, further polymerizing into a stable structure of SeNPs.

The mass ratio of Se/polysaccharide and Se/ascorbic acid, reaction time, and reaction temperature are the most considered parameters regulating the formation of SeNPs. The sequence of factors affecting the particle size of *Grateloupia Livida* polysaccharides-functionalized SeNPs (GLP-SeNPs) was time > temperature > ascorbic acid concentration > polysaccharide concentration according to the R-value. The particle size of GLP-SeNPs becomes larger with the increase in reaction time [28]. Generally, the chemical synthesis of SeNPs was carried out at room temperature. An interesting study was performed to prepare SeNPs at high temperatures (90 °C, 120 °C, 150 °C) by using chitosan (CS) as a reductant agent. The average size of synthesized SeNPs has a strong correlation with the reaction temperature. Although the increasing temperature could accelerate the reaction, the excessively high reaction temperature would promote the intermolecular collision and agglomeration of SeNPs, leading to a larger particle size [29]. Another study pointed out that the increase in the concentration of ascorbic acid enhanced the contact between ascorbic acid and Na_2_SeO_3_, leading to a larger number of SeNPs with lower D_H_ and higher electrostatic stability generated at a shorter reaction time [30]. Among those factors, the mass ratio of Se/polysaccharide has been widely investigated. The peak of SeNPs stabilized by *Cordyceps Sinensis* exopolysaccharide showed a shift from 125 ± 5 nm to 80 ± 3 nm with the Se/P ratio increased from 1:20 to 1:1 but had a reverse shift with the Se/P ratio further increased to 4:3, exhibiting the smallest size particles formed at the Se/P ratio of 1:1. With an initial increase of SeO_3_^2−^, more Se nuclei could be produced by increasing the growth rate of Se, thus facilitating the formation of small-sized SeNPs. Conversely, when the Se/P ratio was beyond 1:1, the formed SeNPs may aggregate together, leading to an increase in the particle size [31]. Pectin (PEC) is an anionic heteropolysaccharide complex that possesses abundant hydroxyl, carboxyl, and ester groups. Se/PEC ratios significantly affected the particle size and surface morphology of PEC-SeNPs, with a Se/PEC ratio of 1:2 exhibiting the smallest mean size of about 41 nm in the aqueous medium [32].

**Figure 3:**
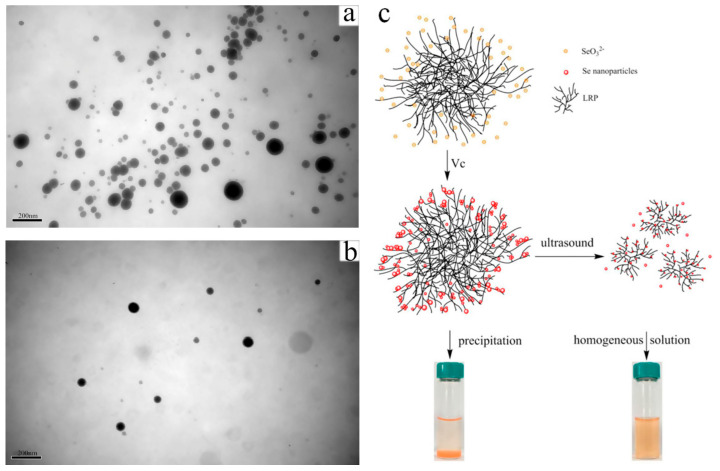
TEM images of LRP-SeNPs (**a**) and U-LRP-SeNPs (**b**) at the Se/LRP ratio of 1/10 in water. Schematic illustration (**c**) for the influence mechanism of ultrasound on LRP-SeNPs [33].

In some cases, assistant techniques such as ultrasonic and microwave have been documented to affect the dispersity and size of SeNPs [14]. The ultrasonic treatment reduced the size of *Lignosus rhinocerotic* polysaccharide functionalized SeNPs (LRP-SeNPs) and narrowed the size distribution. The SeNPs could be easily diffused into the LRP internal branches instead of gathering on the LRP surface and eventually stabilized throughout the extended branches due to the LRP compact coil structure loosened under ultrasonic cavitation (Figure 3) [33]. However, another study reported that the extension of ultrasound time resulted in increased particle sizes, which might be ascribed to the destruction of the structure of stabilizers through enhanced molecular motion by ultrasound, leading to agglomeration of *Oudemansiella radicata* polysaccharide stabilized SeNPs [34]. Hien et al. synthesized SeNPs with a diameter of about 74 nm by gamma Co-60 ray irradiation of H_2_SeO_3_/dextran solution. SeNPs/dextran powder prepared by spray drying technique could be sterilized by gamma Co-60 ray irradiation at a dose of 25 kGy without a noticeable change in SeNPs size [35].

**Figure 4:**
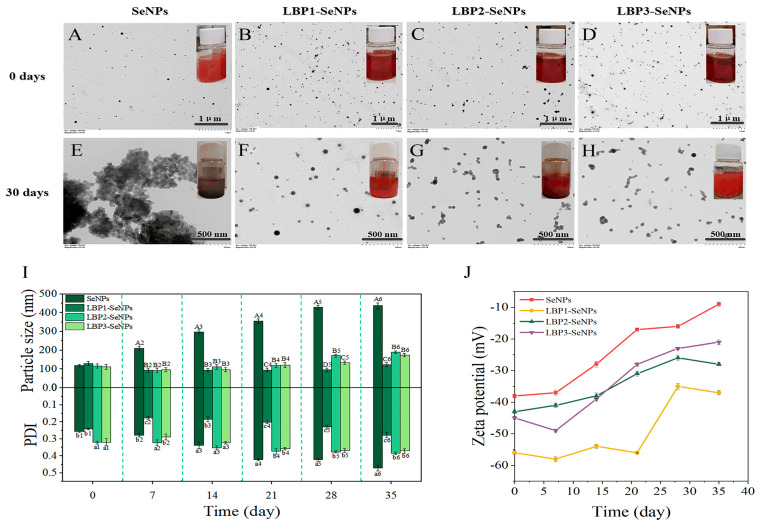
TEM images and optical photograph of SeNPs, LBP1-SeNPs (LBP1: 92,441 Da), LBP2-SeNPs (LBP2: 7714 Da), and LBP3-SeNPs (LBP3: 3188 Da) (**A**–**H**). Particle size and PDI (**I**), and zeta potential (**J**) of SeNPs and LBP-SeNPs during 35 days of storage [36].

The ionized functional groups and the structure of polysaccharides also demonstrated the key influence on the size distribution, stability, and functional activity of SeNPs. Several studies have reported the influence of molecular weight and substituted functional groups of polysaccharides on the morphology and physicochemical properties of SeNPs. The SeNPs prepared with a higher molecular weight of *Lycium barbarum* polysaccharides (LBP, 92,441 Da) exhibited smaller particle size and much better stability than those of SeNPs decorated with a low molecular weight of LBP (7714 and 3188 Da) (Figure 4) [36]. A similar result was also found in the different molecular weights of CS functionalized SeNPs (CS-SeNPs) [37]. However, Zhang et al. found that different molecular weights of CS have a negligible influence on the appearance of CS-SeNPs [38]. Another study reported that carboxylic curdlans (Cur-4, Cur-8, and Cur-24) with different molecular properties (570, 348, 120 kDa) influenced the size of SeNPs, which might also relate to the chain conformations of Cur. Compared with the compact random coils structure of Cur-4, the flexible chains of Cur-8 with decreased molecular weight contribute to the dispersion and stabilization of SeNPs with smaller particle sizes (~56 nm). Excessive carboxylic groups present on the surface of Cur-24 might prevent the SeO_3_^2−^ from capping by intermolecular electrostatic repulsion, leading to the larger SeNPs [39]. It should be pointed out that the superior properties of several polysaccharides obtained by the alkali extraction are limited due to their water insolubility. Molecular modification techniques can be used to modify specific portions of the polysaccharide [40]. A series of water-soluble sulfate-modified polysaccharides (SPS) with different degrees of substitutions (0.02, 0.06, and 0.25) were used as templates to prepare SPS-SeNPs. The results indicated that the size of SPS-SeNPs became smaller and the selenium content was increased with the higher DS of SPS added. This could be ascribed to the higher DS of SPS providing more suitable and stable space for the growing of nano-selenium crystal nuclei, resulting in a stable structure at a smaller size [41]. Moreover, Chen et al. synthesized SeNPs through a wet chemistry method using potassium iodide as a stabilizer and further decorated the surface of SeNPs with positively charged CS and negatively charged carboxymethyl chitosan (CCS) to investigate the effect of ionized functional groups. Their results found that CS-SeNPs exhibited a higher DPPH radical scavenging ability than CCS-SeNPs, which may be due to their hydrogen-donating ability [42].

#### 2.2.2. Protein Functionalized SeNPs

Compared with polysaccharides, proteins possess many advantages as encapsulants or stabilizers including flexible structure, possession of multiple functional groups, amphiphilic nature, and desirable biodegradability [43]. Whereas, few studies have been focused on the fabrication of protein-based SeNPs. It has been reported that bovine serum albumin (BSA)-stabilized SeNPs have highly-ordered superhigh-molecular-weight spherical nanostructures with high density and unique morphology [44]. Meanwhile, BSA stabilized SeNPs exhibited equal efficacy in regulating the activity of glutathione peroxidase and thioredoxin reductase to selenomethionine, but had lower median lethal dose, acute liver injury, and short-term toxicity [45]. It also has been reported that BSA-SeNPs had higher intrinsic oxidase-like activity than sodium alginate (SA)-stabilized SeNPs, but was lower than the positive surface charged CS-SeNPs [46]. However, the particle size and surface properties of BSA-SeNPs could be significantly changed during the oral delivery and influence the absorption in the human body due to the complete digestion of BSA under gastric conditions. Beta-lactoglobulin (Blg) has an isoelectric point (pI) of pH 5.1–5.2, which can be used as a carrier with desirable controlled release properties in the small intestine because of its significant resistance to peptic digestion. SeNPs stabilized by Blg with a mean particle size of 36.8 ± 4.1 nm were stable in acidic (pH 2.5–3.5) or neutral to basic solutions (pH 6.5–8.5) at 4 °C for 30 days. Besides the functional groups of -OH and -NH_2_ which were bonded on the surface of SeNPs, the binding between the hydrophobic domains of Blg and SeNPs as reflected by decreases in protein surface hydrophobicity are also responsible for the stability of SeNPs [47]. However, most proteins have a pI of around pH 4.0~5.0, which restricts their application in the biomedical field. In our recent study, protamine sulfate functionalized SeNPs with an average size of 130 nm were successfully synthesized through physical adsorption, Se-O, and Se-N bonds. The protamine sulfate functionalized SeNPs showed enhanced physical stability at pH varying from 3.0 to 9.0. Compared to bare SeNPs, the positive charge decoration on the surface of SeNPs could improve three-fold cellular uptake efficiency and anticancer activity.

#### 2.2.3. Other Biomolecules Functionalized SeNPs

The biomolecules such as peptides, amino acids, and polyphenols were also investigated as a stabilizer to functionalize SeNPs. Peptides contain many functional groups including -NH_2_, -COOH, and -OH. It was found that the stability of peanut meal peptide mixtures (Ppm)-decorated SeNPs by using ascorbic acid as a reducing agent was better than that of cysteine-decorated SeNPs. Ppm-SeNPs prepared in an ascorbic acid/sodium selenite system with an average particle size of 140 nm have good thermal stability, low-temperature storage stability (stable at 4 °C for 60 days), and pH stability attributed to the self-assembly caused by physical interaction [48]. The formation of tilapia polypeptides (TP)-functionalized SeNPs may induce selenium binding at the expense of the anti-parallel structure in the β-sheet structure of TP. TP-SeNPs were relatively stable in neutral and alkaline environments, with the condition of pH = 8 showing better storage stability, which might be ascribed to the bonding force of electrostatic interaction and hydrophobic interaction [49]. Liao et al. used a walnut peptide to stabilize SeNPs, which demonstrated that SeNPs are effectively capped with walnut peptides via physical absorption, resulting in a stable hybrid structure with an average diameter of 89.22 nm. The peptide binding process in the synthesis of inorganic nanoparticles was highly dependent on the amino acid composition and sequence [50]. Feng et al. fabricated stable and well-dispersed SeNPs by using typical neutral (valine), acidic (aspartic acid), and basic (lysine) amino acids as a decorator. Their results demonstrated that lysine with two -NH_2_ groups and one -COOH group could offer more protonated groups than valine and aspartic acid. Thus, SeNPs@ lysine exhibited better stability, higher cellular uptake efficiency through endocytosis, and higher growth inhibitory effects on various human cancer cell lines [51].

Polyphenols are phenolic hydroxyl group-containing organic molecules, which are widely present in natural plants. They are suitable for fabricating numerous NPs due to their universal adhesive and reducing properties, functional groups, and biocompatibility [52]. Gallic acid (GA), propyl gallate (PG), and pyrogallic acid (PA) have the same pyrogallol structure but different side chains were used as stabilizers to fabricate almost spherical and well-dispersed NanoSe-polyphenol. The average sizes of synthesized NanoSe-polyphenol were 77, 68, and 38 nm, respectively. The ζ-potentials of all nanoparticles were negatively charged at pH 7.4, reaching −19.11, −24.31, and −24.81, respectively [53]. Epigallocatechin-3-gallate (EGCG) can effectively disperse SeNPs at pH 8.0 due to the formation of phenolic anions. However, polyphenol suffered from auto-oxidization and the protonation of EGCG at acidic pH in the stomach causes massive aggregation of SeNPs [54].

**Table 1:** The reaction conditions, size, stability conditions tested, and stability of various biomolecules functionalized SeNPs.

Name	Biomolecules	Reaction Conditions	Size	Stability Conditions Tested	Stability	Ref.
Polysaccharides						
T70-SeNPs	Dextran 70,000	T70: 5 mg/mL, 20 mL; Na_2_SeO_3_: 200 mM, 250 μL; Vc: 200 mM, 1.25 mL; Temperature: 25℃; Time: 12 h	110.3 ± 30.2 nm	Storage stability: 0, 1, 3, 6 months of storage at 4 °C	The freeze-dried powder of T70-SeNPs kept stable for 6 months at 4 °C, while the size of T70-SeNPs solution increased from 117.5 ± 30.4 to 178.5 ± 43 nm in the second month	[55]
PSP-SeNPs	*Polygonatum sibiricum* polysaccharide	PSP: 5 mg/mL; Na_2_SeO_3_: 50 mM, 1 mL; Vc: 200 mM, 1 mL; Temperature: 25 °C; Time: 30 min	114 nm	Storage stability: 0, 1, 5, 10, 20, 30 days of storage at 4 °C; Thermal stability: 25 °C, 50 °C, 70 °C, 90 °C for 1 h; pH stability: pH 2–10 for 1 h; Ionic strength stability: 10, 50, 100 mM NaCl for 1 h	PSP-SeNPs were stable for 20 days of storage at 4 °C and stable at pH 3–10, but easily aggregated in 100 mM NaCl	[56]
ORPS-SeNPs	*Oudemansiella raphanipies* polysaccharide	ORPS: 1 mg/mL; Na_2_SeO_3_: 0.1 M, 0.3 mL; TW-80: 2 mg/mL; Vc: 0.1 M, 1.2 mL; Temperature: 30 °C; Time: 4 h; Dark condition	60 nm	Storage stability: 0, 15, 30, 60, 90, 120 days of storage at 4 °C, 25 °C, 37 °C; pH stability: pH 2–10	ORPS-SeNPs were stable at 4 °C for at least 90 days and stable at pH 4–10	[57]
SPs-SeNPs	*Spirulina platensis* polysaccharide	SPs: 100 mg/mL; Na_2_SeO_3_: 2 mM; Vc: 6 mM; Time: 6 h	73.42 nm	Storage stability: 0, 5, 15, 30, 45, 60, 75, 90 days of storage at 4 °C	SPs-SeNPs were stable for 75 days at 4 °C	[58]
GLP-SeNPs	*Grateloupia Livida* polysaccharide	GLP: 1 mg/mL, 5 mL; Na_2_SeO_3_: 0.01 M, 5 mL; Vc: 0.04 M, 5 mL; Temperature: 45 °C; Time: 3 h	115.54 nm	Storage stability: 0, 5, 10, 15, 20, 25, 30 days of storage at 4 °C and 25 °C	GLP-SeNPs were stable at 4 °C after 30 days of storage and only kept stable for 15 days at 25 °C	[28]
AF1-Se nanocomposite	A highly-branched β-(1→3)-D-glucan	AF1: 1 mg/mL, 100 mL; Na_2_SeO_3_: 0.1 M, 1 mL; Vc: 0.2 M, 2 mL; Temperature: 25 °C; Time: 24 h	92 nm	Storage stability: 1 day and 16 months of storage at room temperature	AF1-Se nanocomposite exhibited excellent stability during 16 months of storage	[59]
PUP-SeNPs	*Polyporus umbellatus* polysaccharide	PUP: 2.5 mg/mL, 0–8 mL; Na_2_SeO_3_: 50 mM, 1 mL; Vc: 20 mM, 10 mL; Temperature: 30 °C; Time: 12 h; Dark condition	82.5 nm	Storage stability: Illumination (dark and 2500 ± 200 Lx), temperature (4 °C, 25°C, 37 °C), time (0–120 days); pH stability: 2–12	PUP-SeNPs possessed good stability at 4 °C in dark conditions for 84 days and were stable at pH 4–12	[60]
CS-SeNPs	Chitosan	CS: 10 mg/mL, 1 mL; H_2_SeO_3_: 20 mM, 1 mL; Vc: 80 mM, 1 mL	387.31 ± 8.13 nm	Storage stability: 0, 5, 10, 15, 20, 25, 30 days of storage at 4 °C; Thermal stability: 30 °C, 50 °C, 70°C, 90 °C for 1 h; pH stability: pH 3–9 for 1 h; Ionic strength stability: 10, 100, 500 mM NaCl for 1 h	CS-SeNPs exhibited good thermal stability and storage stability for 30 days, but easily aggregated in 500 mM NaCl or at pH > 8	[25]
CPP-SeNPs	Fructose-enriched polysaccharide from *Codonopsis pilosula*	CPP: 2 mg/mL, 5 mL; Na_2_SeO_3_: 1.2 M; Vc: 4.8 M; The mass ratio of Na_2_SeO_3_/CPP: 1:20; Temperature: 25 °C; Dark condition	75 nm	Storage stability: 0, 7, 14, 21, 28, 35 days of storage at 4 °C	CPP-SeNPs showed superior stability at 4 °C for at least 35 days	[61]
GLP-SeNPs	Polysaccharides of *Gracilaria lemaneiformis*	GLP: 2 mg/mL; Na_2_SeO_3_: 0.01 M; Vc: 0.04 M; Temperature: 40 °C; Time: 4 h; Dark condition	92.5 nm	Storage stability: 0, 7, 14, 21, 28, 42 days of storage at 4 °C and 25 °C in dark conditions; pH stability: pH 3, 5, 7, 9 for 1 h; Ionic strength stability: 50, 100, 150, 200 mM for 1 h	GLPs-SeNPs were stable at 4 °C under dark conditions for 42 days and kept stable in 50–200 mM ion strengths and at a pH range from 3 to 10	[62]
SFPS-SeNPs	Polysaccharides from *Sargassum fusiforme*	SFPS: 1 mg/mL; Na_2_SeO_3_: 0.01 M; Vc: 0.04 M; Temperature: 50 °C; Time: 4 h	60 nm	Storage stability: 0, 7, 14, 21, 30, 40 days of storage at 4 °C	SFPS-SeNPs remained highly stable at 4 °C for 40 days	[63]
APS-SeNPs	*Astragalus* polysaccharide	APS: 2 mg/mL, 10 mL; Na_2_SeO_3_: 10 mM, 2.4 mL; Vc: 40 mM, 2.4 mL; Temperature: 25 °C; Time: 4 h	62.3 nm	Storage stability: 0, 7, 14, 21, 28, 35 days of storage at 4 °C	APS-SeNPs exhibited good stability for 35 days at 4 °C	[64]
PEC-SeNPs	Pectin	PEC: 1 mg/mL, 200 mL; Na_2_SeO_3_: 0.1 M; Vc: 0.2 M, 1 mL; Temperature: 25 °C; Time: 24 h	41 nm	Storage stability: 0, 5, 10, 15, 20, 25, 30 days of storage at 4 °C pH stability: pH 3, 4, and 5 during the storage time (0–30 days)	PEC-SeNPs were highly stable at pH > 4.0 for at least 1 month	[32]
SeNPs-C/C	Chitosan/citrate gel	CS: 10 mg/mL; Na_2_SeO_3_: 40 mg/mL; Vc: 40 mg/mL	1–30 μm	Storage stability: 0, 5, 10 days of storage at 60 ± 1 °C, 80 ± 5% RH, and 5000 ± 500 Lx (Stress testing); 1, 2, 3, 6 months storage at 40 ± 2 °C, 75 ± 5% RH, dark (Accelerated testing)	SeNPs-C/C exhibited excellent stability after 6 months of storage in a simulated package environment (40 ± 2 °C, 75 ± 5% RH, dark)	[65]
Proteins						
Blg-SeNPs	Beta-lactoglobulin	Blg: 10 mg/mL, 1 mL; Na_2_SeO_3_: 0.06 M, 1 mL; Vc: 0.3 M, 5 mL; Time: 30 min	36.8 nm	Storage stability: 0, 30 days of storage at 4 °C or 25 ± 1 °C; pH stability: pH 2.5, 3.5, 4.5, 5.5, 6.5, 7.5, 8.5	Blg-SeNPs were stable in acidic or neutral to basic solutions (pH 2.5–3.5 or 6.5–8.5) at 4 °C for 30 days	[47]
BSINPs	Bovine serum albumin	BSA: 20.63 mg/mL, 40 mL; Na_2_SeO_3_: 20 mM, 10 mL; Glutathione: 25 mM, 40 mL; Time: 1 h	40 nm	Storage stability: 0, 1, 2, 3, 4 weeks of storage	BSINPs remained around the initial particle size without aggregation or precipitation over 4 weeks	[66]
Other biomolecules						
EWP-SeNPs	Egg white polypeptide	EWP: 35 mg/mL; Na_2_SeO_3_: 1.038 mg/mL; Temperature: 82 °C; Time: 3.5 h	30–50 nm	Storage stability: 0, 8, 30 days of storage at 4 °C; pH stability: pH 2, 4, 6, 8, 10 for 8 and 30 days	EWP-SeNPs showed excellent stability in an alkaline environment (pH = 10) for 30 days at 4 °C	[67]
TP-SeNPs	Tilapia polypeptide	TP: 20 mg/mL; Na_2_SeO_3_: 0.692 mg/mL; Temperature: 50 °C; Time: 21 h	200 nm	Storage stability: 0, 8 days of storage at 4 °C; pH stability: pH 2, 4, 6, 8, 10 for 8 days at 4 °C	TP-SeNPs were relatively stable in an alkaline environment (pH = 8) after 8 days of storage at 4 °C	[49]
Ppm-SeNPs	Peanut meal peptides mixture	Ppm: 3–4 mg/mL; Na_2_SeO_3_: 1 mM; Vc: 4 mM; Temperature: 55℃; Time: 6 h	140 nm	Storage stability: Two months of storage at 4 °C and 25 ± 1 °C; Thermal stability: 90 °C for 1, 3, and 6 h; pH stability: pH 2, 6, 10 for 2 weeks at 4 °C	Ppm-SeNPs exhibited good thermal stability and alkali resistance, and were stable for 60 days at 4 °C	[48]
PSP-SeNPs	Polysaccharide-protein complex	PSP: 0.08%; Na_2_SeO_3_: 1 mM; Vc: 4 mM; Temperature: 25 °C	63.33 nm	Storage stability: 0, 4, 8, 12 months of storage at 4 °C	PSP-SeNPs remained stable for 12 months at 4 °C	[68]
Bc@SeNPs	Betacyanins	Bc: 14 mg/mL, 5 mL; Na_2_SeO_3_: 7 mg/mL, 5 mL; Vc: 30 mg/mL, 5 mL; Temperature: 4 °C; Time: 24 h	133 nm	Storage stability: 0, 5, 10, 15, 20, 25, 30 days of storage at 4 °C	Bc@SeNPs maintained good stability in an aqueous solution at 4 °C for 30 days	[69]

### 2.3. The Stability of Functionalized SeNPs

The stability of nanoparticles is a key factor affecting their applications. The surface functionalization with biomolecules leads to the formation of a stable core-shell nanostructure, which could protect the energy-saturated surface of SeNPs and increase their stabilization in an aqueous solution. Most research found that SeNPs decorated with polysaccharides or proteins could increase the stability of NPs from day one to the range of 15–60 days at 4 °C [70,71,72]. An interesting study was conducted by using highly-branched β-(1→3)-D-glucan (AF1) self-assembled hollow nanofibers as a stabilizer to synthesize highly stable SeNPs. The results found that SeNPs with an average size of 46 nm were entrapped in the cavities of the AF1 hollow nanofibers through the formation of Se-O bonds, leading to the excellent stability of AF1-SeNPs for at least 16 months of storage [59].

**Figure 5:**
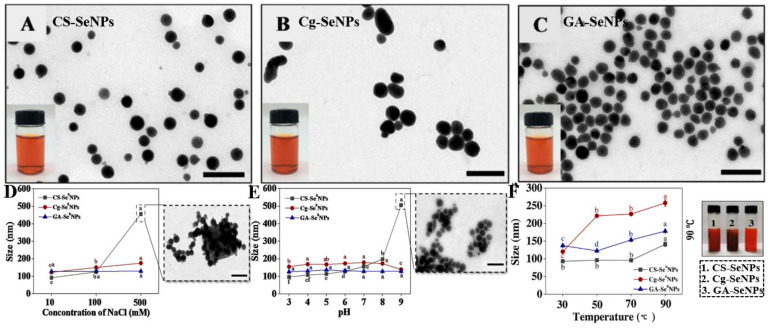
Microstructure and optical photograph of CS-SeNPs (**A**), Cg-SeNPs (**B**), and GA-SeNPs (**C**) investigated by TEM. Effect of different ionic strengths (**D**), pH (**E**), and temperature (**F**) on the stability of CS-SeNPs, Cg-SeNPs, and GA-SeNPs [25].

However, during the storage process, the stability and functionality of SeNPs could be strongly affected by various environmental factors including temperature, ionic strength, and pH. The average size became larger and the nanoparticles were aggregated into clusters at higher storage temperatures. Compared to carrageenan (Cg) and CS, gum arabic (GA) with a multi-branched structure provided better thermal stability of functionalized SeNPs. Positively charged CS-SeNPs were easily aggregated in high ionic strength at 500 mM NaCl due to the shield of electrostatic interactions induced by salt ions. Interestingly, although the ζ-potential of negatively charged Cg-SeNPs was decreased due to the binding of Na^+^ to κ-carrageenan double helices, it did not result in the aggregation of NPs (Figure 5). The effect of pH on the stability of SeNPs mainly depends on the pKa value of the ionizable functional groups [25]. Cationic nanoparticles had a favorable affinity with the anionic cell membrane, but they had a disappointingly short time in blood circulation [73]. It found that the particle size of CS-SeNPs was slightly increased when the pH values increased from 3 to 8 and the nanoparticles occurred aggregation as the pH further increased to 9. This phenomenon might be ascribed to the reduced surface charge and decreased solubility of CS. In contrast, the particle size of Cg-SeNPs and GA-SeNPs exhibited no significant change at any pH, which is more stable at a wider range of pH than CS-SeNPs [25]. Moreover, the processing treatments such as heating, freeze-drying-rehydration, and freeze-thawing could affect the physicochemical and functional properties of NPs. Song et al. found that all the above processing treatments could cause CS-SeNPs aggregation [74].

Although surface functionalization could improve the stability of SeNPs in an aqueous solution at 4 °C, it is still challenging to maintain a colloidal SeNPs solution for a long time at room temperature. Moreover, it is not convenient for the storage or transportation of SeNPs at low temperatures. Thus, several strategies have been developed to preserve SeNPs. For instance, cross-linking and ionotropic gelation could be used to further improve the stability of SeNPs. Reversible ionic gelation between CS and citrate has been applied to load SeNPs in the presence of CS (SeNPs C/C). Subsphaeroidal SeNPs C/C microspheres of 1–30 μm were obtained by spay-drying and were very stable at high temperature, high humidity, and strong light. Especially, SeNPs C/C microspheres exhibited excellent stability in a simulated package environment (40 ± 2 °C, 75 ± 5% RH, dark), which remained the original appearance after 6 months of storage and could be easily released [65]. Cavalu et al. used alginate and alginate/CS microspheres incorporating SeNPs through cross-linking and ionotropic gelation. SeNPs were mainly exposed on the surface of alginate or laced in a porous alginate/CS matrix with a tri-dimensional structure. The microspheres as carriers could effectively prolong the duration of selenium release in SIF with a minimized release in SGF [75]. Moreover, transforming the aqueous solution into powder also becomes a suitable method for improving the stability of SeNPs. It was reported that the particle size of SeNPs/Oligochitosan (OCS) powder prepared by spray drying was slightly increased to 43.8 nm as compared to the SeNPs/OCS solution with an average size of 41.8 nm [76]. Particularly, the particle sizes of SeNPs/β-Glucan powder increased from 92 nm in the solution to 95, 117.6, and 132.3 nm prepared by freeze-drying, coagulation, and spray drying techniques, respectively, indicating that the freeze-drying method is the best way to preserve SeNPs [77].

## 3. Targeting Strategies for SeNPs

Although the above strategies could improve the stability of SeNPs, the rapid elimination from the circulatory system, low cellular accumulation, systemic toxicity, and side effects are still the main barriers to the actual application of SeNPs in cancer therapy [78]. The unique surface character and excellent anticancer activity of SeNPs make it easy to be modified with targeted ligands to improve the targeting efficiency and lower the side effects. Thus, targeted SeNPs have emerged as more efficient chemotherapeutic agents in cancer therapy. Herein, we summarize the current research on tumor-targeting strategies for the application of SeNPs.

### 3.1. Achieving Targeting by Avoiding Reticuloendothelial System (RES)

Various types of nanoparticles have been demonstrated to accumulate in solid tumors through the enhanced EPR effect [79]. The morphology, hydrodynamic size, surface charge, as well as the nanoparticles’ constituent materials are the major factors that influence cellular uptake, resulting in various biological activities [80]. It should be pointed out that the reticuloendothelial system (RES) blocks the functionality of nanoparticles due to the adsorption of serum proteins such as immunoglobulins (Ig) and complement proteins on the surface of nanoparticles that enhance the macrophage recognition and phagocytosis, leading to the removal of nanoparticles [81].

The development of strategies to prolong the duration of the blood circulation time of SeNPs has become urgent. Nanoparticles with a hydrophilic surface have the potential to repel plasma proteins. For example, polyethylene glycol (PEG) is an electrically neutral, highly biocompatible, and hydrophilic molecule, which is also listed as “Generally Recognized as Safe” (GRAS) by FDA. Due to its unique characteristics, PEG could not only prevent the interaction between SeNPs and blood components, reducing the uptake by the macrophages of the mononuclear phagocyte system (MPS) but also play a key role in the formation of stable hybrid SeNPs. The morphology characters could be regulated by varying the concentration or molecular weight of PEG, with the increased PEG concentration leading to higher hydrophilization on the surface of SeNPs [82]. Another study constructed selenium nanosystems by using polyvinylpyrrolidone (PVP), poly-N, N, N-trimethylacry-loyloxyethylammonium methylsulfate (PTMAEM), and PEG as stabilizers, demonstrating the role of nature and molecular weight of the polymer on the thermodynamic characteristics [83]. Monodisperse PEG-SeNPs with ultrasmall diameter were formed ascribed to the interaction between oxygen and selenium atoms, as well as the amphoteric properties of PEG. Further investigations found that PEG-SeNPs exhibited stronger growth inhibition on drug-resistant hepatocellular carcinoma cells through induction of mitochondria dysfunction [84]. Although several studies have reported the synthesis of stabilized SeNPs using PEG, little research has investigated whether the decoration of PEG could prolong the blood circulation time or not.

The red blood cell membrane (RBCM) possesses unique characteristics, including high biocompatibility, long circulating half-life, flexible and stable membrane, non-tumorigenicity, and large superficial surface area conducive to surface modification, which also proved to evade immunogenic clearance [85,86]. Lin et al. used natural RBCM to construct a biomimetic multifunctional Ru-complex-functionalized SeNPs. In vitro experiments demonstrated that RBCM endows Ru-complex-functionalized SeNPs with the ability to escape the phagocytosis of macrophages [87]. In another study, a highly hemocompatible erythrocyte membrane-coated ultrasmall selenium nanosystem was designed. As expected, the RBCM coating effectively prolonged the blood circulation time and reduced the elimination of the nanosystem by the autoimmune response. This nanosystem also demonstrated potent anticancer activity towards A375 cells without any obvious histological damage to the non-target major organs [88]. Compared to the PEGylated nanosystem, RBCM-functionalized nanoparticles significantly reduce the immunogenicity and prolong the blood retention time.

### 3.2. Tumor-Activated Targeting

**Figure 6:**
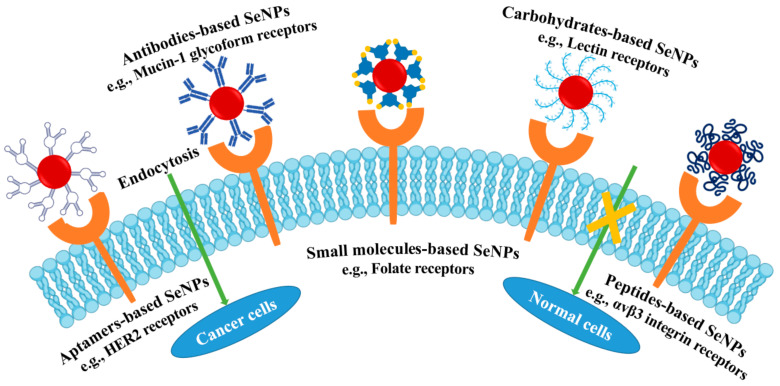
Various targeting ligands functionalized SeNPs.

Without the incorporation of targeting ligands, nanoparticles passively accumulated in cancer through the enhanced EPR effect, which was not sufficient for targeting therapeutics to tumors. Thus, a broad range of targeting ligands such as small molecules, carbohydrates, antibodies, peptides, and aptamers have been explored to functionalize the surface of NPs to achieve targeting properties (Figure 6) [89]. Various ligand-conjugated SeNPs with different endocytosis and their potential biochemical mechanism were presented in Table 2.

#### 3.2.1. Small Molecules-Based Targeting SeNPs

Small molecules with infinitely diverse structures and properties have great potential as targeting moieties. FA, a water-soluble vitamin B9, is one of the most extensively utilized targeting ligands. It is essential for the biosynthesis of DNA, cell division, and cell growth, especially during embryonic development. The folate receptors (FR) have overexpressed on the surface of various human carcinomas including breast, lung, kidney, and so on. Therefore, FA-conjugated nanoparticles have a high binding affinity to FR, which enables the targeting delivery of NPs [90]. It has been reported that FA-SeNPs with an average diameter of 70 nm were internalized by MCF-7 cells through the FR-mediated endocytosis way. The IC_50_ value of FA-SeNPs for MCF-7 cells was 2.47 μg/mL, while lower cytotoxicity was observed in normal CBMSC cells with an IC_50_ value of 22.52 μg/mL. The formed endocytic vesicles transported FA-SeNPs into the cytoplasm. Then, FA-SeNPs accumulated in mitochondria and entered the nucleus, further inducing the mitochondria-dependent apoptosis and causing damage to the nucleolus by arresting the cell cycle [91]. Further study on the uptake mechanism revealed that the FR-mediated endocytosis way of the FA-SeNPs in MDAMB231 and A375 cells involved clathrin as well as caveolin-mediated endocytosis. FA-SeNPs along with MEK inhibitor (PD98) demonstrated a synergistic antiproliferative efficacy, which can induce cell apoptosis through the cell cycle arrest, caspase-3 activation, reactive oxygen species (ROS) production, and mitochondrial depolarization, combined with the inhibition of the MAPK pathway [92].

Folate targeting achieved through carbodiimide conjugation of FA to polymers has also been documented. Maiyo et al. synthesized stable and well-dispersed CS-functionalized FA-targeted SeNPs for the delivery of mRNA. Compared to the cells with less or no FR, the nanoparticles showed significant transgene expression in the overexpressed FR-positive KB cells [93]. Their further investigations prove the ligand-receptor interactions with a high binding energy of −4.4 kcal mol^−1^ between SeChFA and the receptor FOLR1 by molecular docking studies [94]. Folic acid-gallic acid-N, N, N-trimethyl CS (FA-GA-TMC) was also designed as a stabilizer to fabricate cubic-like SeNPs. In this case, the positive charge from -N^+^ (CH_3_)_3_ of the quaternized CS increased the electrostatic interaction between the negatively charged SeNPs and the stabilizer. The hydrophobic groups of GA and FA could offer the π-π stacking interactions and hydrogen bonding interactions to regulate the formation of SeNPs. Furthermore, SeNPS@FA-GA-TMC exhibited a higher IC_50_ value (15.41 μM) than SeNPs@Vc (74.20 μM) and showed no cytotoxicity toward BT474 cells. It was associated with the decoration of GA and FA on the surface of SeNPs that enhanced the cell permeability in biological processes and specifically targeted the overexpressed folate receptor on the surface of cancer cells [95]. The modified folic acid-N-trimethyl CS (TMC-FA)-stabilized SeNPs (SeNPs@TMC-FA) were also reported as pH-responsive nanocarriers for the delivery of DOX to overcome multidrug resistance. The DOX-SeNPs@TMC-FA exhibited 10-fold higher cytotoxicity as compared to free DOX, which also demonstrated a higher cumulative release of DOX under the acidic condition at pH 5.2 (54.1%) within 2 h than the release at pH 7.4 (12.3%) [96]. Xia et al. prepared SeNPs by decorating them with polyethyleneimine (PEI), linked with FA as a tumor-targeting siRNA delivery (Se-PEI-FA@siRNA). The Se-PEI-FA@siRNA entered HepG2 cells through clathrin-mediated endocytosis and exerted antitumor efficacy by inhibiting protein expression of CDK2, cyclin E, and cyclin D1, and up-regulating the protein expression of p21 [97].

Lactobionic acid (LA) is a multifunctional galactosylation molecule with unique characteristics such as biocompatibility, biodegradability, and self-assembly properties. Galactose (GA) is a widely explored ligand for targeting cells due to the abundance of lectin receptors on the surface of cells [98]. Both LA and GA are considered as ideal candidates for targeting hepatocytes and liver cancer cells due to the high binding affinity with the asialoglycoprotein receptor (ASGPR) present in the above cells [99]. One recent study was carried out to prepare LA-polyethylene glycol-CS-functionalized SeNPs (LA-PEG-CS-SeNPs) for ASGPR targeting on HepG2 cells. The results indicated that LA-PEG-CS-SeNPs were primarily taken up by ASGPR-mediated endocytosis with the highest transgene expression in HepG2 cells than Caco-2 and HEK293 which do not have the ASGPR [100]. Shaigan et al. also found similar results in poly-L-lysine-lactobionic acid-capped SeNPs, which exhibited significant transgene expression in HepG2 cells [101]. GA-modified SeNPs (GA-SeNPs) also showed excellent cellular uptake efficiency by entering HepG2 cells through the clathrin-mediated endocytosis pathway. The active targeting delivery system GA-Se@DOX also exhibited higher anticancer activity than the passive targeting delivery system, showing no significant damage to major organs [102].

Triphenylphosphine (TPP) is a well-established functional organic moiety with significant mitochondria-targeting properties. Mitochondria are one of the most critical organelles, playing a key role in the energy supply, calcium homeostasis regulation, and signal transduction of cancer cells [103]. Mitochondria are also on the verge of oxidative stress, considered as key regulators of the apoptosis pathway [104]. Thus, mitochondrial targeting is an effective strategy for the treatment of cancer via precise and severe damage to mitochondria. Zhang et al. designed mitochondria-targeting SeNPs coated with HAS and TPP. The mitochondria-targeting efficiency was found to be proportional to the number of TPP moieties. TPP modification significantly increased the colocalization of SeNPs on mitochondria, leading to enhanced ROS production, efficient mitochondrion damage, and higher anticancer activity [105].

#### 3.2.2. Carbohydrate-Based Targeting SeNPs

Carbohydrates play a key role in many cellular processes that are important for life [106]. At present, carbohydrates have been reported as active-targeting glycosyl ligands due to the selectivity recognization by the cell surface receptors [107]. Hyaluronic acid (HA) is a ligand for the cluster of differentiation 44 (CD44). HA has been used as a stabilizer to prepare SeNPs (HA-SeNPs), exhibiting a high tumor inhibition ratio of 49.12% in vivo at a relatively low dose of 4.32 mg/kg, whereas the positive group treated with 5-Fu (25 mg/kg) showed an inhibition ratio at 61.71% [108]. The tumor-targeted HA-Se@DOX exhibited selective cellular uptake between cervical cancer HeLa cells and human umbilical vein endothelial cells (HUVEC). It could trigger HeLa cell apoptosis via activating the Bcl-2 signaling pathway [109]. *Gracilaria lemaneiformi* polysaccharide (GLP) has been reported as an important receptor of cellular adhesion molecules, possessing a high affinity to αvβ3 integrin in the majority of malignant tumors. Jiang et al. used GLP as an integrin-targeting surface decorator to synthesize SeNPs. Their results found that GLP-SeNPs exhibited higher cellular uptake in U87 cells with high integrin expression levels than in C6 cells with low integrin expression levels [110]. Lentinan (LNT) could inhibit the growth of cancer cells by targeting toll-like receptor-4 (TLR4). The LNT functionalized SeNPs could target the mitochondria of tumor cells via the TLR4/TRAF3/MFN1 through caveolae-mediated endocytosis [111].

#### 3.2.3. Peptides-Based Targeting SeNPs

Peptides present many advantages, including low immunogenicity, biocompatibility, high drug loading capacities, chemical diversity, and ease of manufacture at low cost. They also provide targeted recognition with high affinity, high specificity, high penetration, and rapid excretion ability [112]. The αvβ3 and αvβ5 integrin are overexpressed by tumor endothelial cells, which could be specifically recognized by peptide motif Arg-Gly-Asp (RGD). Thus, RGD-containing peptides have been identified as potential tumor-targeting peptides. A previous study found that the surface decoration of RGD could significantly enhance the internalization of SeNPs through receptor-mediated endocytosis and induce apoptosis in HUVECs cells via suppression of VEGF-VEGFR2-ERK/AKT signaling. RGD-SeNPs have been proved to be used as an efficient carrier to achieve cancer-targeted antiangiogenesis synergism due to the bioresponsive triggered drug release under acidic conditions [113]. Xia et al. carried out a series of studies to synthesize SeNPs using a positively charged cyclic peptide (Arg-Gly-Asp-d-Phe-Cys, RGDfC) as a targeting molecule. They confirmed the effective accumulation of RGDfC-conjugated SeNPs in the tumor in vivo and demonstrated the specific uptake in HeLa cervical cancer cells via clathrin-associated endocytosis. Their results also showed the potential of RGDfC-conjugated SeNPs to be used as carriers of anticancer drugs such as siRNA and DOX [114,115,116]. The synthetic peptide GE11 with the sequence of Y-H-W-Y-G-Y-T-P-Q-N-V-I has been proved to be an efficient epidermal growth factor receptor (EGFR) targeting peptide. Pi et al. found that EGFR-targeted SeNPs decorated by GE11 and oridonin (GE11-Ori-SeNPs) exhibited enhanced cellular uptake efficiency through EGFR-mediated endocytosis and showed active targeting effects in a nude mice xenograft model. GE11-Ori-SeNPs could induce cell apoptosis by inhibiting EGFR-mediated PI3K/AKT [117].

A single-target point is prone to off-target effects. Hence, the development of dual-targeting nanomaterials has attracted great interest. TGN and LRFFD are the peptides that could specifically recognize receptors on the blood-brain barrier (BBB) and selectively bind to a particular hydrophobic domain in Aβ_40_ aggregates. Yang et al. designed two targeting peptides (LPFFD and TGN) conjugated SeNPs and found that the dual-functionalized SeNPs can cross the BBB and have a strong affinity toward Aβ species to inhibit Aβ aggregation [118]. It has been reported that uPA can be used as a target for uPAR which is highly expressed in HCC cells. The matrix metalloproteinase MMP-2/MMP-9 ACPP can cut ACPP to expose the positively charged segment. Thus, uPA and ACPP were connected to CS via an amide bond and further used as a decorator to prepare dual-targeting modified SeNPs (u/A-SeNPs) through electrostatic interaction. The u/A-SeNPs not only improved the stability of nanoparticles in the physiological environment but also exert higher antitumor activity, which could increase levels of autophagy [119].

#### 3.2.4. Antibodies-Based Targeting SeNPs

**Figure 7:**
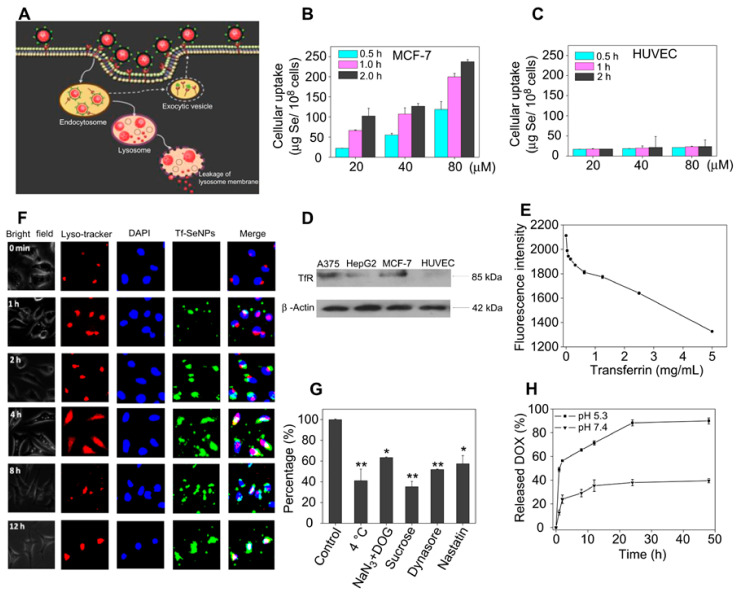
Proposed endocytosis pathway of Tf-SeNPs in MCF-7 cells (**A**). Quantitative analysis of cellular uptake efficacy of coumarin-6-loaded Tf-SeNPs in MCF-7 cells (**B**) and HUVEC cells (**C**). TfR expression in A375, HepG2, MCF-7, and HUVEC cells (**D**). Free Tf competed with coumarin-6-loaded Tf-SeNPs for binding to TfRs on MCF-7 cells (**E**). Intracellular trafficking of coumarin-6-loaded Tf-SeNPs (**F**). Intracellular uptake of Tf-SeNPs in MCF-7 cells under different endocytosis-inhibited conditions. Difference with *p* < 0.05 (*) or *p* < 0.01 (**) was considered statistically significant as compared to the control group (**G**). In vitro release profiles of DOX from Tf-SeNPs in PBS solution (pH 7.4 and pH 5.3) (**H**) [125].

Antibodies are typically 200–300 nm in size proteins known as immunoglobulins. They have gained growing attention in biopharmaceutical research because of their therapeutic functionality and targeting properties [120]. Human epidermal growth factor receptor-2 (HER2 antigen) is overexpressed in cancer cells and plays a pivotal role in cell growth, migration, differentiation, and survival. One major strategy for HER-2 targeting is to use antibodies due to their inherent affinity and specificity [121]. Liu et al. employed an HER-2 antibody with a disulfide linkage as a surface decorator to fabricate SeNPs (HER2@NPs) for precise drug delivery. The HER2@NPs were internalized by receptor-mediated endocytosis through the dynamin-related lipid raft-mediated and clathrin-mediated pathways. Meanwhile, the HER2@NPs could induce the apoptosis of human bladder cancer cells at nontoxic concentrations and enhance anticancer efficacy in vivo [122]. Another study used the HER2 antibody-conjugated SeNPs for the effective delivery of SPIONs into the brain. The results indicated that HER2 functionalization could overcome the BBB efficiently and precisely deliver the SPIONs to brain tissues [123]. Anti-transferrin receptor monoclonal antibody (OX26) surface decoration was also proved to enhance the targeted transport of SeNPs through transferrin receptor-mediated endocytosis and could contribute to neuronal survival by targeting different cellular signaling pathways [124]. In a similar case, the transferrin (Tf)-conjugated SeNPs exhibited the Tf-guided selectivity between cancer and normal cells due to the high expression levels of TfR in MCF-7 cancer cells. The results found that Tf-SeNPs showed 1–6-folds higher cellular uptake efficiency in MCF-7 cells than HUVEC cells after 0.5, 1.0, 2.0 h of incubation with 80 μM of Tf-SeNPs, which could enter cells through clathrin-mediated and caveolae/lipid raft-mediated endocytosis (Figure 7) [125].

#### 3.2.5. Aptamers-Based Targeting SeNPs

Aptamers are small nucleic acid ligands that can bind their cognate targets with high affinity and specificity. Compared to antibodies, aptamers exhibited low or no immunogenicity and possessed high stability under a wide range of temperatures and storage conditions [126]. The SeNPs modified with aptamers showed good chemical stability, good biocompatibility, and low toxicity, which could efficiently target HEp-2 cells with overexpressed nucleolin (NCL). Moreover, the results indicated that the aptamers modified SeNPs could be used as a good light scattering nanoprobe in dark-field microscopy (DFM) imaging [127]. Jalalian et al. developed epirubicin (EPI)-loaded-NAS-24-functionalized PEI-PEG-5TR1 aptamer coated SeNPs (ENPPASe complex). The ENPPASe complex exhibited good internalization into MCF7 (human breast carcinoma cell) and C26 (murine colon carcinoma cell) cancer cells using 5TR1 aptamer as the target agent, as reflected by the fluorescence imaging and flow cytometry analysis, which also proved to significantly reduce the toxicity in non-target cells [128].

**Table 2:** Various ligand conjugated SeNPs with different endocytosis and their potential biochemical mechanism.

Name	Ligand	Receptors	Endocytosis Mechanism	Biochemical Mechanism	Ref.
Small molecules-based					
FA-SeNPs	Folic acid	Folate receptors	Clathrin and caveolin-mediated endocytosis	ROS overproduction and mitochondrial depolarization	[92]
SeNPs@TMC-FA	Folic acid	Folate receptors	Folate receptor-mediated endocytosis	Regulation of caspase-3 and PARP	[96]
LA-PLL-SeNPs	Lactobionic acid	ASGPR	ASGPR-mediated endocytosis	N/A	[101]
Carbohydrate-based					
GA-SeNPs	Galactose	Lectin receptors	Clathrin-mediated endocytosis	Activating caspase signaling and Bcl-2 family proteins	[102]
GLP-SeNPs	*Gracilaria lemaneiformis* polysaccharide	αvβ3 integrin	αvβ3 integrin-mediated endocytosis	Downregulation of intracellular reactive oxygen species and activation of p53, MAPKs, and AKT pathways	[110]
LNT-SeNPs	lentinan	TLR4/TNF receptor	Caveolae-mediated endocytosis	Regulation of mitochondrial membrane fusion pathway, mediated by factor 3 (TRAF3)/mitofusin-1 (MFN1) protein complex	[111]
Peptides-based					
RGD-NPs	RGD peptide	αvβ3 integrin receptor	αvβ3 integrin-mediated endocytosis	Induction of apoptosis and cell cycle arrest in HUVECs via suppression of VEGF-VEGFR2-ERK/AKT signaling axis	[113]
GE11-Se NPs	GE11 peptide	EGFR	Lipid raft-mediated and clathrin-mediated endocytic pathway	Induction of reactive oxygen species production, activation of the mitochondria-dependent pathway, and inhibition of EGFR-mediated PI3K/AKT, and Ras/Raf/MEK/ERK pathways	[117]
Antibodies-based					
HER2@NP	HER2 antibody	HER2 receptors	Receptor-mediated endocytosis	Triggered DNA damage-mediated p53 signaling pathways	[123]
OX26-PEG-Se NPs	Monoclonal antibody (OX26)	Anti-transferrin receptor	Transferrin receptor-mediated endocytosis	Regulation of cellular metabolic state (TSC1/TSC2, p-mTOR, mTORC1), oxidative defense system (FoxO1, β-catenin/Wnt, Yap1), inflammatory reactions (jak2/stat3, Adamts-1), autophagy and apoptotic cell death (Mst1, ULK1, Bax, caspase-3 and Bcl-2)	[124]
Aptamers-based					
PEI-PEG-5TR1 aptamer coated SeNPs	5TR1 aptamer	Mucin-1-glycoform	MUC1 receptor-mediated endocytosis.	N/A	[128]

## 4. SeNPs as Delivery Vehicles

SeNPs have demonstrated potential anticancer activity in cancer chemotherapy, as reflected in liver cancer, breast cancer, ovarian cancer, colon cancer, skin cancer, and prostate cancer [8]. In brief, studies on the mechanism of the anti-tumor effect revealed that SeNPs inhibited cell proliferation by generating the ROS through the mitochondrial-mediated pathway, arresting the cell cycle, initiating apoptosis by the activation of the p53 and MAPK pathway, as well as regulating proteins such as Bcl-2 family and caspase-3 family proteins, which are related to apoptosis [129,130]. Besides the excellent anticancer activity, the high surface areas and tunable surface structures of SeNPs enables them to effectively load drugs or genes through covalent or non-covalent interaction. Thus, SeNPs also have been appointed as potential anticancer drug or gene delivery carriers with higher delivery efficacy, synergistic effect, and low toxicity, which could overcome severe adverse effects induced by conventional chemotherapeutic drugs or gene on normal cells [131]. Several functionalized SeNPs applied for single or co-delivery of drugs and genes are summarized in Table 3.

### 4.1. Single Delivery

**Figure 8:**
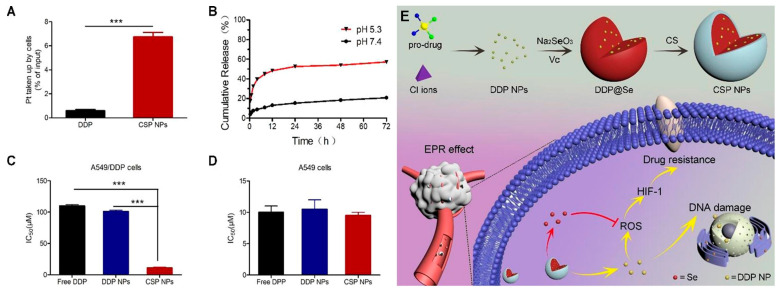
The quantitative intracellular uptake of free DDP and the CS-coated Se/DDP nanoparticles (CSP NPs) (**A**). pH-responsive release of CSP NPs. (**B**). The IC_50_ values of free DDP, DDP NPs, and CSP NPs against A549/DDP and A549 cells (**C**,**D**). The design and function of CSP NPs (**E**). *** *p* < 0.001 indicated statistically significant [133].

Conventional drugs are unfortunately limited by their poor solubility, high toxicity, and short circulating half-lives, which represent the greatest challenges in cancer therapy [139]. Resistance to drugs also has become the main cause of death in many diseases. SeNPs have been used as carriers to deliver DOX, 5FU, temozolomide (TMZ), cisplatin (DDP), and other drugs with promising results. Liu et al. hypothesized designing a synergistic system using SeNPs as a carrier of 5FU. The SeNPs were connected to 5FU through physical adsorption and covalent interaction by the formation of Se-O and Se-N bonds. The in vivo and in vitro studies indicated that 5FU-SeNPs showed a high anti-tumor effect with IC_50_ values ranging from 6.2 to 14.4 μM to five human cancer cell lines, which could inhibit the growth of cancer cells through caspase-dependent apoptosis [140]. Yu et al. developed a facile pH-assisted method for preparing curcumin-functionalized SeNPs (SeNPs@Cur) through physical adsorption and the formation of Se-O bonds. The SeNPs@Cur possess high drug conjugation efficiency of up to 5.5% and enhanced solubility of Cur [141]. The SeNPs@Cur inhibited cell proliferation by activating caspase-3 mediated apoptosis via P53 and AKT signaling pathways [142]. Liu et al. reported the delivery of sesamol using polyethylene-glycol-functionalized SeNPs. The loading efficiency of sesamol reached 10.0 ± 0.5 wt % and the seamol-PEG-SeNPs showed higher apoptosis than sesamol or PEG-SeNPs alone, which was ascribed to the downregulation of Bcl-2 and procaspase-3, upregulation of Bax and PARP, and discharge of cytochrome c into the cytosol [135]. The SeNPs coated with CS and Eudragit^®^ RS100 (Eud) were prepared for TMZ delivery to a TMZ-resistant cell line (C6 cell line). The Se@TMZ/Eud-Cs exhibited a high loading efficiency up to 82.77% ± 5.30 with pH-sensitive release kinetics. The expression of the main genes including MGMT and RELA in drug resistance was significantly decreased as compared to the free form of TMZ [143]. A similar study was carried out to design CS-SeNPs as nano-vehicles for the encapsulation of DDP to combat HIF-1-induced drug resistance. CS-SeNPs significantly enhanced the cellular uptake of DDP and the release rate at pH 5.3, which reached 50% at 12 h while less than 15% of DDP was released in pH 7.4. The results verified that the nanocarrier could inhibit two drug-resistance pathways including ROS-HIF-1-GCLM-GSH and ROS-HIF-1-MDR-MRP2/P-gp, further reversing the side effects of treatment-induced ROS in HIF-1 activation and cisplatin-resistance (Figure 8) [133]. In addition, BSA-SeNPs were also reported as nanocarriers of indocyanine green (ICG) to achieve highly effective chemo-photothermal combination therapy. The results indicated that the nanocarrier significantly enhanced the stability of ICG and displayed a higher temperature response under laser irradiation than free ICG, which could synergistically induce U87L cell death and completely suppressed U87L tumor growth [66].

The surface decoration of SeNPs with various ligands could further enhance the selectivity and anticancer efficacy of drugs [144]. The cancer-targeted FA-SeNPs were developed to deliver RuPOP, which increased the selectivity efficiency to FA receptor overexpressed cancer cells and against the multidrug-resistant by inhibition of ABC family proteins expression. Moreover, the FA-SeNPs induced cell apoptosis by triggering MAPKs and AKT pathways by up-regulating the level of ROS [145]. A modified folic acid-N-trimethyl CS (TMC-FA) stabilized SeNPs was used as nanocarriers for the delivery of DOX to overcome drug-resistant cancer cells. The pH-responsive SeNPs enhanced the anticancer efficacy of DOX through the apoptosis way by regulating caspase-3 and PARP proteins, which exhibited a 10-fold reduced IC_50_ value compared to free DOX [96]. To overcome the low cellular uptake of paclitaxel (PTX) in cancer cells, HA was linked with SeNPs to fabricate a tumor-targeted delivery vehicle. The HA-Se@PTX exhibited an acid pH-dependent PTX release feature in the microenvironment of cancer cells, resulting in great anti-tumor activity in vivo [132].

In recent years, gene therapy by using plasmid DNA (pDNA) and siRNA has gained momentum due to their immense potential for application in cancer vaccination and immunotherapy. However, the instability and susceptibility to degradation have become a great challenge. SeNPs have also been widely used as gene carriers for delivering to the target site. Pillay et al. formulated dendrimer functionalized SeNPs (PAMAM-SeNPs) containing FA as targeted moiety for delivery of pDNA. The FA-conjugated PAMAM-SeNPs with desired size (<150 nm) and ζ-potentials (>25 mV) successfully protected the pDNA from degradation in an in vivo stimulation and exhibited higher transgene expression in Hela cells [146]. It has been reported that siRNA holds advantages with transient expression over DNA transfection, making it more attractive in applications [93]. To construct an active targeting siRNA delivery system, SeNPs were successfully prepared by using the RGDfC peptide as the capping agent. The results indicated that SeNPs decorated with RGDfC peptide could efficiently encapsulate siRNA and deliver siRNA into the tumor site, leading to significant Oct4 gene silencing in HepG2 cells. The RGDfC-SeNPs/siRNA could induce apoptosis by regulating the Wnt/β-catenin signaling pathway and activating the BCl-2 signaling pathway [114]. Although recent studies have confirmed the synergistic effects of SeNPs in a combination of siRNA for targeting gene silencing, the SeNPs delivery systems developed still presented the limitation of the stability and protection of siRNA. Sharifiaghdam et al. synthesized a stable Se-based siRNA-loaded nano-complex (NCs) using a straightforward layer-by-layer self-assembly method. The LBL-Se-NCs possess a sandwich structure: the core (Se@CS) with a second layer of the cargo (siRNA), and the third layer of CS. The third layer ensured the stability of siRNA with only 35% release in 7 days compared to CS-NCs, which occurred with 100% release of siRNA after 48 h. Their further research designed highly stable selenium-based layer-by-layer (LBL) nano-complexes (NCs) with polyethyleneimine (PEI-LBL-NCs) as the final polymer layer for siRNA delivery [136]. PEI-LBL-NCs presented good protection of siRNA with only 40% siRNA release in a buffer of pH = 8.5 after 72 h or in simulated wound fluid after 4 h. However, siRNA was completely released in CS-LBL-NCs under the same condition. This might be explained by the fact that PEI had a broader proton capacity in the pH range from 3 to 10 [147].

### 4.2. Co-Delivery

Multidrug resistance (MDR) is a major barrier to cancer chemotherapy. One important strategy for overcoming MDR is the combination of drug-based therapy and gene-based therapy. The versatility of nanocarriers provides a platform for binding and transporting different therapeutic cargos. Recently, several studies were conducted using SeNPs as carriers for co-delivery. Fang et al. used RC-12 and PG-6 peptides as specific cancer-targeting ligands to functionalize SeNPs and further designed to carry DOX and ICG with the loading capacity of 15.35 ± 1.2% and 13.20 ± 2.1% to achieve the combination of chemotherapy and photothermal therapy. The results indicated that the DOX release ratio reached 82.5% for pH 5.3 and 36% for pH 7.4, and the ICG accumulated release ratio was 70.7% for pH 5.3 and 35.0% for pH 7.4 under the near-infrared (NIR) light laser irradiation, whereas the release ratio of DOX and ICG without NIR laser irradiation was much lower. The synthesized SeNPs-DOX-ICG-RP could induce a rapid increase in ROS and promote the death of HepG2 cells, resulting in highly efficient anticancer activity by a combination of chemo- and photothermal therapy [138]. Multifunctional polyamidoamine-modified SeNPs were also designed for the dual-delivery of siRNA and cisplatin. The co-localization of siRNA and cisplatin in A549/DDP cells could overcome drug resistance by arresting cells at the G1 phase and inducing apoptosis via the MAPKs and PI3K/AKT signaling pathways. Moreover, the nanocarriers combined with chemotherapy and gene therapy exhibited no appreciable abnormality in the major organs [137]. Another study was carried out by using mannose-modified CS-modified SeNPs to co-deliver ampicillin (Ap) and β-lactamase inhibitor. The co-delivery of ampicillin and β-lactamase inhibitor could effectively inhibit β-lactamase activity and enhance the anti-infective efficiency by inhibiting inflammatory response, inducing cell apoptosis, and regulating multiple signal pathways [148].

## 5. Conclusions and Perspective

In recent years, we have witnessed enormous progress in the construction of multifunctional nanomaterials. To overcome the narrow quantitative range of the dose between anticancer efficacy and toxicity of inorganic selenium (e.g., sodium selenite and sodium selenate), nanotechnology has been applied for the development of novel SeNPs. Numerous pieces of research have demonstrated the low toxicity and high anticancer activity of SeNPs. In this review, we summarized the various strategies for the functionalization of SeNPs with biomolecules to achieve increased stability. The size, morphology, surface charge, and stability of SeNPs were associated with the mass ratio of Se/decorator, reaction time, reaction temperature, and functional groups, as well as molecular weight, and chain conformation of biomolecules. The drying methods could further affect the stability of SeNPs. Further functionalized SeNPs with various ligands demonstrated a strong interaction between the nanoparticles and receptors expressed in cancer cells, resulting in higher cellular uptake efficiency through receptor-mediated endocytosis and targeted ability to enhance the anticancer activity. It also has the potential as nanocarriers for the single or dual delivery of chemotherapeutic drugs and gene drugs. Moreover, the anticancer mechanism of SeNPs differs according to the targeting moieties used.

Although significant progress has been made in the design of functionalized SeNPs in the past few years, some challenges are still present for further application:

(i) Surface functionalization could improve the storage stability of SeNPs. However, the stability under the complex microenvironment of the tumor should be further investigated. For instance, the interaction of SeNPs with serum protein or macrophages.

(ii) Stimuli-responsive SeNPs using a hierarchical targeting strategy should be designed to overcome the complex physicological environment and achieve a higher anticancer activity.

(iii) The biodistribution and the relationship of structure-bioactivity of functionalized SeNPs should be better understood.

(iv) The synergic activity between the SeNPs and other chemotherapeutic drugs needs to be clarified.

(v) A more robust processing, manufacturing, and regulatory infrastructure need to be established. In addition, more toxicity evaluations including long-term cytotoxicity and genotoxicity, in vivo animal and pre-clinical studies should be carried out for expediting their clinical translation.

## Figures and Tables

**Table 3:** Functionalized SeNPs as nanocarriers for drug/gene delivery.

Drug/Gene Loaded	Nanocarrier	Release Properties	Effects	Ref.
Doxorubicin	Folic acid-N-trimethyl chitosan stabilized SeNPs	The release rate was 12.3% and 54.1% at pH 7.4 and 5.3 within 2 h	Induce cell death through the apoptosis pathway by the involvement of caspase-3 and PARP proteins.	[96]
Paclitaxel	Hyaluronic acid-modified SeNPs	The release rates were 45.7% and 59.4% in pH 7.4 and 6.8	Activate the caspase-3-related apoptosis pathway	[132]
Cisplatin	Chitosan-coated SeNPs	The release rates reached 50% at 12 h in pH 5.3 while less than 15% was released at pH 7.4	Reduce ROS levels to prevent HIF-1 activation	[133]
Curcumin	Pectin-decorated SeNPs	The cumulative release was 60% at pH 3.0 and 17% at pH 7.0 within 8 h	Inhibit the growth of HepG2 cells	[134]
Seamol	Polyethylene-glycol-functionalized SeNPs	The release rate reached 65.4% at 24 h and 84.7% at 48 h (pH 5.4)	Induce apoptosis by down-regulating of Bcl-2 and procaspase-3, up-regulating Bax and PARP	[135]
siRNA	Layer-by-layer Se-based nanocomplexes	The release rate was only 35% after 7 days	Induce around 32% apoptosis in H1299 cancer cells	[136]
siRNA/cisplatin	Amine-terminated generation 5 polyamidoamine dendrimers-modified SeNPs	The accumulated siRNA released rate reached 80%	Induce apoptosis involving the AKT and ERK signaling pathways	[137]
Doxorubicin/ indocyanine green	RC-12 and PG-6 peptides functionalized SeNPs	The release ratio was 82.5% for pH 5.3 and 36% for pH 7.4 with NIR laser irradiation	Induce apoptosis by triggering ROS overproduction	[138]

## Data Availability

Not applicable.
